# Relationship between community physical activity levels, social capital, intergenerational support, and subjective well-being among older adults in China: a moderated mediation model

**DOI:** 10.3389/fpubh.2025.1520565

**Published:** 2025-04-03

**Authors:** Yuanzheng Lin, Changxu La, Qingyuan Luo, Peng Zhang

**Affiliations:** ^1^College of Physical Education and Health Science, Yibin University, Yibin, China; ^2^School of Wushu, Chengdu Sport University, Chengdu, China; ^3^College of Physical Education and Sports, Beijing Normal University, Beijing, China

**Keywords:** community physical activity, subjective well-being, social capital, intergenerational support, older adults

## Abstract

**Objective:**

This study investigates the mediating role of social capital in the effect of community physical activity on older adults’ subjective well-being and the moderating role of intergenerational support.

**Methods:**

Questionnaire data were collected from 318 older adults from China who participated in community-based physical activity using a random sampling method with the Physical Activity Scale, the Social Capital Scale, the Intergenerational Support Scale, and the Subjective Well-Being Scale. Data were analyzed by SPSS 25.0, PROCESS 3.4 and AMOS 24.

**Results:**

Community physical activity positively affected older adults’ subjective well-being (β = 0.539, *p* < 0.001), and social capital mediated the effect of community physical activity on older adults’ subjective well-being (β = 0.162, *p* < 0.001, 23.11% of the total effect). Intergenerational support moderated the relationship between community physical activity and older adults’ subjective well-being (β = 0.133, *p* < 0.001).

**Conclusion:**

Active participation in community physical activity can directly improve the subjective well-being of older persons on the one hand, and indirectly improve well-being by increasing social capital on the other. In addition, good intergenerational relationships can lead to more active participation in community physical activity, which in turn affects the subjective well-being of older people.

## 1 Introduction

China is experiencing rapid population aging, with individuals aged 65 and above projected to constitute over 20% of the population by 2033 (1). This demographic shift poses significant challenges to the nation’s health and social systems, particularly as many older adults face unmet health and emotional needs. Addressing these challenges requires innovative approaches to promote healthy aging and enhance the well-being of older adults. Community physical activity has garnered attention as a means to improve physical health and foster social connections among older adults (2–4). However, the mechanisms through which these activities influence subjective well-being remain unclear. Social capital, characterized by trust, reciprocity, and social networks, may play a critical mediating role by strengthening interpersonal relationships and reducing feelings of isolation (5). Furthermore, in Chinese society, where intergenerational support is a culturally embedded value, its moderating effect on the benefits of physical activity warrants deeper investigation.

Despite extensive research on the physical benefits of exercise, there is limited understanding of how community physical activity interacts with social and familial factors to shape well-being in aging populations, particularly in the unique cultural context of China. This study seeks to address this gap by exploring (1) the direct effects of community physical activity on subjective well-being, (2) the mediating role of social capital, and (3) the moderating effect of intergenerational support. By examining these dimensions, this research aims to contribute to both theoretical understanding and practical solutions for promoting healthy aging in Chinese communities.

## 2 Literature review and research hypothesis

### 2.1 Community physical activity and subjective well-being

Physical activities have returned to their original prosperity after the pandemic in China, older people’s participation in physical activity mainly takes place in the community, which makes them the main force of community physical activities. On the one hand, participation in community physical activity enables older people to maintain their physical health, and on the other hand, it also promotes their psychological health so that they can spend their later years better. Activity theory suggests that high-frequency social interactions contribute to the emotional well-being of older adults, that interpersonal contact is essential for maintaining self-awareness, and that people can derive meaning from the roles they play (6). Older adults with high levels of social interaction, especially in informal groups, are more likely to find meaningful relationships and therefore have higher levels of emotional well-being. Current studies have shown that physical activity in the community promotes mental health and improves quality of life for older adults (7, 8), and has a strong positive association with their well-being (9). Participation in physical activity by older adults has a positive effect on alleviating mental health problems such as anxiety and depression (10), and chemicals such as endorphins released through exercise help to elevate their emotional state (11). In particular, collective forms of physical activity in the community are more capable of strengthening the emotional ties of older groups and promoting their emotional expression (12). In addition, physical activity can also enhance the self-esteem of the older adult, and by overcoming the difficulty of sports and making progress, the older adult can feel a sense of accomplishment and enhance their sense of identity, thus improving their subjective well-being (13).

Based on this, this study proposes Hypothesis 1: Community physical activity positively affect the subjective well-being of older adults.

### 2.2 The mediating role of social capital

Robison (14) define social capital as “the sympathy that an individual or group feels for another person or group, which may result in benefits and preferences that go beyond the expected exchange relationship.” Social–emotional selection theory tells us that older people’s social relationships decrease with age, and they value meaningful socialization more than younger people because meaningful socialization can promote their emotional well-being (15), and that community-based physical activity provides older adults with opportunities to interact and connect, promoting the accumulation of social capital (e.g., trust, belonging, and reciprocal behaviors). Robison et al. note that empathy and emotional connection are foundational to social capital formation, and that these elements can be reinforced through community physical activity. Older adults develop a need to cooperate in community physical activities due to common goals, and this need promotes the generation of empathy and trust, as well as strengthens the emotional bond between people (16). According to Maslow’s Hierarchy of Needs theory, older adults crave a sense of belonging and emotional support, and social capital helps fulfill these needs by promoting trust and interaction, enhancing older adults’ sense of well-being (17). The interpersonal circles established through community physical activities can enhance human communication and interaction, giving people more opportunities to meet people from different industries and obtain better quality heterogeneous social resources (18). Participation in community physical activities can lead to more harmony, trust and solidarity in the neighborhoods of older people, and has a significant effect on promoting their self-identity (19). With high levels of trust, interaction and social interaction among community residents, more social capital is also accumulated, making it easier to develop a positive and energizing atmosphere from which people are more likely to gain emotional connectivity, which in turn enhances their personal well-being (20).

Based on this, the study proposed Hypothesis 2: Social capital mediates the role of community physical activity in influencing the subjective well-being of older adults.

### 2.3 The moderating role of intergenerational support

Intergenerational relationships are one of the most important factors affecting the well-being of older adults and a necessary condition for healthy aging. Under the influence of the Chinese culture of “raising children for old age” and “filial piety,” more older people expect their children to support them when they reach adulthood, so intergenerational support from children is particularly important for older people’s old age, and those who receive more support from their children will experience less depression, loneliness and malnutrition, and vice versa (18). Financial support and daily care provided by children has a positive effect on the physical and mental health of older adults (21), the higher the level of intergenerational support provided by children, the better the mental health of older adults (22), and the receipt of intergenerational support significantly enhances the subjective well-being of older adults (23, 24). It has been found that children’s support mainly affects older adults’ subjective well-being by influencing their sense of self-esteem, loneliness, and sense of beneficence (25). Although children are an important factor influencing the life satisfaction of older adults, studies have shown that this influence is not dependent on the number of people, but rather on the ability of children to provide more emotional and behavioral support to their parents (26). It can be seen that in Chinese family relationships, children’s support has a significant positive impact on older people’s well-being in later life.

Based on this, this study proposes Hypothesis 3: Intergenerational support has a moderating role in the process of the effect of community physical activity on the subjective well-being of older adults.

### 2.4 Hypotheses and conceptual model

**Figure 1:**
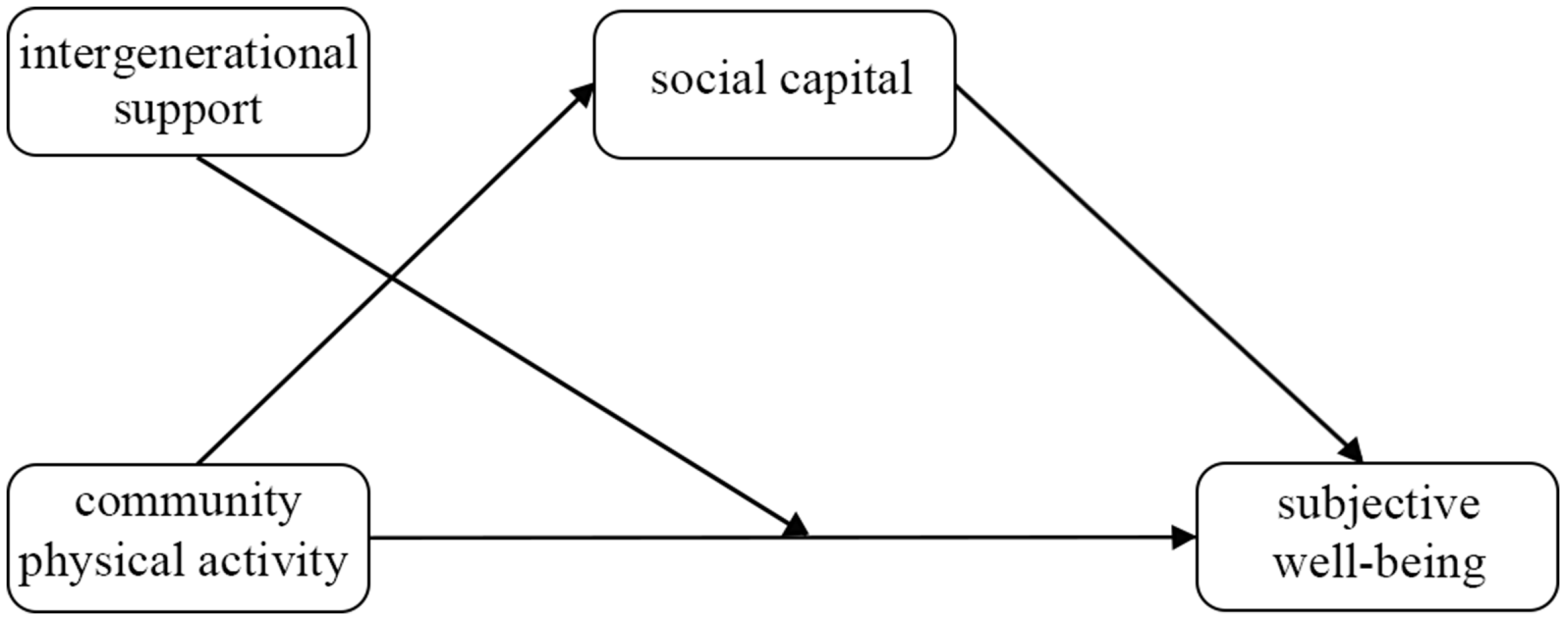
The hypothetical model.

The hypothetical model of this study is shown in Figure 1, where community physical activity directly affects subjective well-being, social capital mediates the effect of community physical activity on subjective well-being, and intergenerational support moderates the effect of community physical activity on subjective well-being.

## 3 Materials and methods

### 3.1 Participants and procedure

Using random sampling method to conduct online and offline surveys on older adult people participating in sports activities from multiple communities. Validated scales with high reliability were used to measure all constructs. Prior to data collection, the survey was pre-tested with 30 participants to ensure cultural relevance and clarity. The online procedure for distributing the questionnaire was: by contacting the administrators or core participants of community sports, the questionnaire was designed through Questionnaire Star, distributed to WeChat groups, and subjects completed their responses on their cell phones. Subjects who lacked exercise once a week, responded within 1 min, repeated IPs, and duplicate accounts were excluded, which prevented invalid or duplicate submissions. Offline, trained investigators conducted face-to-face interviews according to standardized protocols and monitored to ensure consistency. Ultimately, a total of 318 older adults with normal cognitive function and no significant physical or mental illness volunteered to participate in the study. To minimize bias, participants were assured of confidentiality, and questions were neutrally phrased. Data entry was double-checked, and discrepancies were resolved through consultation with the data collectors. Ethical approval was obtained, and all participants provided written informed consent.

### 3.2 Instruments

#### 3.2.1 Community physical activity

In this study, the participation of older adults in community physical activity was measured using Liang’s Physical Activity Rating Scale (27), which has very good reliability and validity in testing individuals’ level of physical activity participation and has been adopted by many researchers in China. The specific way to measure the exercise level of the older adult is by scoring the time, frequency and intensity of their participation in community physical activity, the scoring method is as follows: the score range of the exercise time is 0–4 points, the score range of the frequency and intensity of the exercise is 1–5 points, the level of participation in the exercise = time*frequency*intensity, with the lowest score being 0 points and the highest score being 100 points, and finally, according to the composite scores of the three dimensions The level of exercise was assessed, with higher scores indicating higher levels of participation in community physical activity. In the present study, the Cronbach’s alpha coefficient was 0.822.

#### 3.2.2 Social capital

Robison (14) elaborated on the commonalities between social capital and other forms of capital (e.g., physical capital, human capital), especially the attributes of transformation capacity, durability, flexibility, decay and maintenance, which provide a new perspective and theoretical basis for the study of social capital. Robison pointed out that social capital has the basic characteristics of capital, and that defining it as empathy helps to better understand its role. Thus, social capital is not just a collection of social relationships, but also a “resource” that can produce real benefits. For example, the fact that someone receives an opportunity or support as a result of empathy and trust not only demonstrates the value of relationships, but also reveals a deeper connection between emotions and economic behavior. Based on the research of Robison et al. and referencing Liu’s (28) research, a social capital scale was designed, with the questions “In general social interactions/contacts that do not directly involve monetary benefits, do you feel that there are many people you can trust among the friends you exercise with?,” “Do you and your friends exercise together? The questions were “How familiar are you and your friends who exercise with each other” and “Do you and your friends who exercise with you support each other in your daily life?” Answer items were assigned a value of 1–5 in descending order. In the present study, the Cronbach’s alpha coefficient was 0.881.

#### 3.2.3 Intergenerational support

According to Wang et al. (29) and Yin et al. (30), intergenerational support was divided into three dimensions: economic support, life care, and emotional support. For example, the question of economic support is “the status of financial support given by children every month,” and the answer is assigned by “none = 1, low = 2, medium = 3, high = 4, and very high = 5”; and the question of life care is “the feeling of children taking care of themselves.” The question on life care was “How well do children take care of themselves,” and responses were assigned by “none = 1, low = 2, medium = 3, high = 4, very high = 5”; the question on emotional support was “How well does intergenerational communication take place,” and responses were assigned by “Almost no contact (less than once a year), less than once a month (more than once every 2 months), at least once a month (1–3 times/month), at least once a week (1–4 times/week), almost every day (5–7 times/week) “, assigned values 1 to 5, respectively. In the present study, the Cronbach’s alpha coefficient was 0.873.

#### 3.2.4 Subjective well-being

Subjective well-being has developed greatly over the past few decades, however, some current studies have only tested general well-being when studying well-being, i.e., the question was designed as “Overall, do you feel happy now?,” which has some limitations in measurement. Therefore, this study adopted Inglehart’s protocol for measuring subjective well-being variables, using happiness and life satisfaction to produce an SWB index (31). Happiness is self-reported happiness, with five options from “very unhappy” to “very happy,” assigned values from 1 to 5; Life Satisfaction is life satisfaction, which involves the respondents’ family financial situation, interpersonal relationships, personal health, and personal health. Life Satisfaction is life satisfaction, which involves the respondents’ satisfaction in eight aspects, such as family economic situation, interpersonal relationship, personal health, housing situation, the community they live in, work and life, etc., with five options ranging from “very dissatisfied” to “very satisfied” and assigned a value from 1 to 5. In the present study, the Cronbach’s alpha coefficient was 0.913.

### 3.3 Control variables

Subjective well-being is a psychological state contributed to by many factors, and in addition to the main explanatory variables, there is a need to control for other important explanatory variables in the study. In this study, variables of demographic socio-economic characteristics such as gender, age, education, marital status, and income are used as control variables.

### 3.4 Analysis

The data for this study were organized and analyzed using SPSS 25.0, AMOS 24 and the SPSS macro program PROCESS 3.4 developed by Hayes. First, the common method bias problem was tested by Harman’s one-way test, which showed that the 1st one-way explanatory variance from the unrotated exploratory factor analysis was 27.33% (<40%), so there was no serious method bias problem in this study. Next, the collected samples were subjected to reliability tests and regression analyses, validated factor analyses of the structural equations, and Pearson correlation analyses were used to determine the relationships between the variables. Then, we performed mediation and moderating effects tests by Bootstrap method. Finally, slope plots were drawn to enable us to visualize the effects of the moderating variables on the individual paths in a more intuitive manner.

## 4 Results

### 4.1 Descriptive statistics and correlations among the main study variables

Descriptive statistics and correlation analysis are crucial steps in understanding and interpreting data in research. Table 1 presents a description of the characteristics of the participants, including: age, gender, education, marital status and income. The study sample consists mainly of females (57.55%), with the majority aged 60–70 years (80.19%). The education level is relatively low, as 59.12% have only a primary school education. In terms of marital status, 93.71% are married, indicating strong family stability. Income levels are generally low, with 68.24% earning less than 3,000 RMB per month.

**Table 1:** Demographic characteristics of the samples.

Variable	Frequency	Percentage (%)
Gender		
Male	135	42.45%
Female	183	57.55%
Age		
60–65	140	44.03%
66–70	115	36.16%
71–75	47	14.78%
75<	16	5.03%
Education		
Primary school	188	59.12%
High school	81	25.47%
College and higher	49	15.41%
Marital status		
Married	298	93.71%
Unmarried	0	0.00%
Divorced	9	2.83%
Widowed	11	3.46%
Income		
<2,000	87	27.36%
2,001–3,000	130	40.88%
3,001–4,000	64	20.13%
4,001–5,000	22	6.92%
5,000<	15	4.72%

Table 2 shows the mean and SD values for each variable, as well as the Pearson correlations between the variables. The results showed that community physical activity and subjective well-being of older adults were significantly and positively correlated with social capital; social capital was significantly and positively correlated with the subjective well-being of older adults; and intergenerational support was significantly and positively correlated with the other variables.

**Table 2:** Descriptive statistics and correlations for primary variables.

Variable	M	SD	1	2	3	4
1 Community physical activity	2.73	1.04	1			
2 Social capital	3.25	0.97	0.803^***^	1		
3 Intergenerational support	3.04	0.69	0.374^***^	0.315^***^	1	
4 Subjective well-being	3.15	1.03	0.706^***^	0.639^***^	0.503^***^	1

*p < 0.05; **p < 0.01; ***p < 0.001.

### 4.2 The test of reliability and validity

Prior to the analysis of the structural equations, we conducted a validation factor analysis of each variable using AMOS 24 to ensure that the question items for each scale were consistent with our research predictions. In the study by Chain (32), a value of CR greater than 0.7 and a value of AVE greater than 0.5 were considered to be the ideal criteria. In our study, the compositional reliability among the variables ranged from 0.823–0.913, and the mean extracted variance ranged from 0.560–0.636 (Table 3), indicating that these variables have good reliability and validity. In addition, we also analyzed the goodness-of-fit of the structural equation model using AMOS 24, in which GFI, AGFI, CFI, NFI and IFI were all greater than 0.9, and RMSEA = 0.033 (Table 4), with reference to Wu (33), the indexes of the structural equation model’s goodness-of-fit reached the standard, and we can judge that our model is more reliable.

**Table 3:** Validity and reliability test of the questionnaires.

Variable	CR	AVE
Community physical activity	0.822	0.607
Social capital	0.882	0.560
Intergenerational support	0.874	0.580
Subjective well-being	0.912	0.635

CR, composite reliability; AVE, average variance extracted.

**Table 4:** Model fit indices.

	*χ*^2^/df	*p*	GFI	AGFI	CFI	NFI	IFI	RMSEA
Indices	1.342	0	0.939	0.920	0.986	0.949	0.987	0.033

### 4.3 The mediation model analysis

According to the results in Table 5, community physical activity positively predicted subjective well-being (β = 0.539, *p* < 0.001), and hypothesis 1 was supported; community physical activity positively predicted social capital (β = 0.745, *p* < 0.001), and social capital positively predicted subjective well-being (β = 0.217, *p* < 0.001). In addition, Table 6 shows that the value of the direct effect of community physical activity on subjective well-being was 0.539, with a 95% confidence interval of [0.410, 0.668], and the value of the indirect effect of social capital was 0.162, with a 95% confidence interval of [0.044, 0.281], and the 95% confidence intervals for the direct and indirect effects did not contain 0, indicating that community physical activity had both the direct and indirect effects were significant, social capital mediated the effect of community physical activity on subjective well-being, and hypothesis 2 was supported.

**Table 5:** Regression analysis of the mediation model.

Predictors	Step 1 (Social capital)			Step 2 (Subjective well-being)		
	β	SE	*t*	β	SE	*t*
Community physical activity	0.745	0.031	23.94 ***	0.539	0.036	14.972 ***
Social capital				0.217	0.032	6.781 ***
*R* ^2^	0.645			0.513		
*F*	272.967 ***			165.566 ***		

***p < 0.001.

**Table 6:** Bootstrapping analysis of the mediation model.

	Effect	SE	95% CI	Ratio to Total Effect
Direct Effect	0.539	0.036	[0.410, 0.668]	76.89%
Indirect effect	0.162	0.033	[0.044, 0.281]	23.11%
Total effect	0.701	0.028	[0.623, 0.779]	-

### 4.4 The moderating model analysis

As shown in Table 7, the model was further tested for mediation and moderating effects. Community physical activity positively predicted older adults’ subjective well-being (β = 0.229, *p* < 0.001), validating Hypothesis 1. Community physical activity positively predicted social capital (β = 0.745, *p* < 0.001), and social capital positively predicted older adults’ subjective well-being (β = 0.222, *p* < 0.001), supporting Hypothesis 2. Through the interaction term between community physical activity and intergenerational support, it positively predicted older adults’ subjective well-being (β = 0.133, *p* < 0.001), suggesting that intergenerational support moderated the effect of community physical activity on older adults’ subjective well-being, and Hypothesis 3 was supported.

**Table 7:** Regression analysis of the mediation model.

Variable	M: Social capital			Y: Subjective well-being		
	β	SE	*t*	β	SE	*t*
Constant	3.249	0.042	77.357 ***	2.378	0.045	52.844 ***
Community physical activity	0.745	0.031	23.937 ***	0.229	0.031	7.387 ***
Social capital				0.222	0.035	6.343 ***
Intergenerational support				0.381	0.031	10.886 ***
Community physical activity × Intergenerational support				0.133	0.032	4.156 ***
*R* ^2^	0.645			0.592		
*F*	272.967 ***			113.913 ***		

*p < 0.05; **p < 0.01; ***p < 0.001.

**Figure 2:**
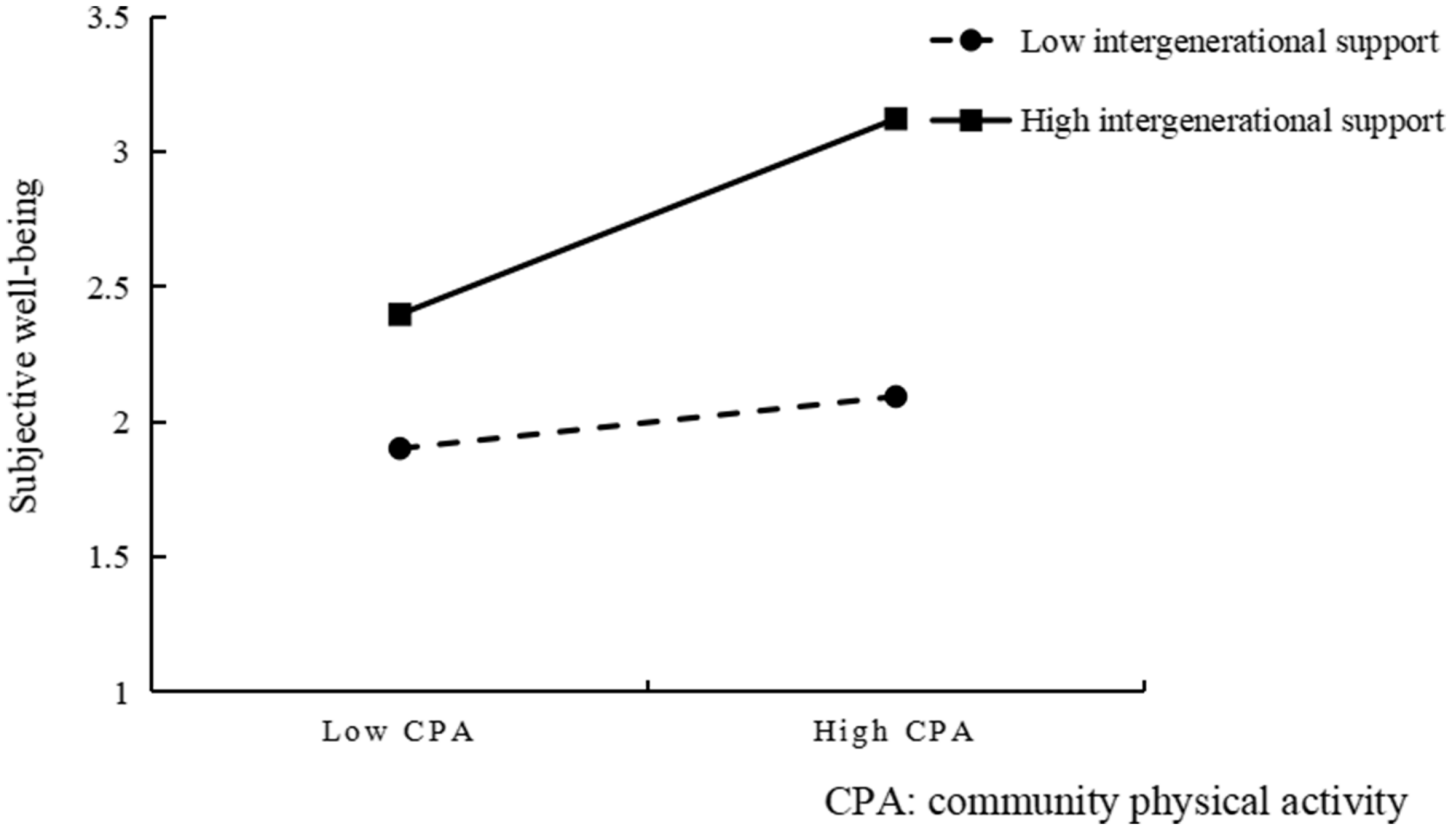
Interaction between community physical activity and intergenerational support.

In order to present a clearer picture of the moderating effect of intergenerational support, a simple slope test was performed by grouping the intergenerational support variables according to the criterion of one standard deviation above and below the mean, as suggested by Preacher et al. (34). As can be seen from Figure 2, At lower levels of intergenerational support, the positive predictive effect of community physical activity on the subjective well-being of older adults is significant, and at higher levels of intergenerational support, the positive predictive effect of community physical activity on the subjective well-being of older adults is strengthened, indicating that the positive predictive effect of community physical activity on the subjective well-being of older adults becomes stronger gradually as the level of intergenerational support increases.

## 5 Discussion

Subjective well-being among older adults in China is facing significant challenges due to societal transformations, including urbanization and the weakening of traditional family support systems (35). Many older people experience loneliness, reduced life satisfaction and diminished quality of life (36), which has become a serious public health concern. Older adults adjust to declines in social roles, physical health, and social support, leading to lower self-esteem and well-being (37). This study demonstrates that community physical activity can directly improve subjective well-being and indirectly enhance it through the accumulation of social capital. By fostering trust, reciprocity, and supportive social networks, these activities address emotional and social needs, alleviating feelings of isolation. Furthermore, improving subjective well-being aligns with broader public health goals, contributing to healthier aging and reducing the societal and economic burden of an aging population. These findings highlight the urgent need for policies and programs that prioritize community engagement and social connection among older adults.

### 5.1 The direct effect of community physical activity

Older adults will directly promote their subjective well-being by participating in physical activity in the community, and this study verified the results of previous research (7–9). Physical health is an important factor affecting the subjective well-being of older adults, and physical health is significantly correlated with older adults’ well-being, and older adults maintain a healthy body, which is an important source of their well-being (38). As in other studies, when we consider community physical activity as a form of physical activity, its positive impact on the physical and mental health of older people is significant, which has been confirmed in many studies (39). It has been proved that older people are more prone to physical and psychological illnesses, such as various chronic diseases, depression, anxiety, and loneliness, all of which have a significant negative effect on their well-being (40). By participating in community physical activity, on the one hand, it can improve the physical activity level of the older adult and reduce the probability of physical diseases; on the other hand, community physical activity can encourage the older adult in the community to communicate with each other, express their inner feelings, and satisfy their self-emotional needs, which can further reduce the occurrence of psychological diseases such as anxiety and depression (41, 42). Different from previous studies on sports participation, this paper investigates the participation of older adults in the community in physical activity, emphasizes the form of multi-person exercise in community physical activities, and further validates the positive impact of community physical activity on the subjective well-being of older adults. As communities are the main venue for the daily activities of older persons, the Chinese Government should now actively promote the construction of public services for community sports, create fitness points for all, and guide older persons to participate in community physical activity, in order to build an informal community support network and to promote a sense of well-being among older adults.

### 5.2 The mediating role of social capital

In this study, social capital mediates the effect of community physical activity on the subjective well-being of older adults. The results suggest that older people’s participation in community physical activity not only directly increases their level of well-being, but also indirectly improves their subjective well-being by increasing their social capital. This mechanism of action is similar to the idea proposed by Robison et al. (17) that social capital in emotional exchanges (e.g., empathy, trust) can produce “socio-emotional goods,” which satisfy basic human caring and belonging. Specifically, community sport is not only a facilitator of physical health, but also an important vehicle for the accumulation of social capital (43). Physical activity not only strengthens “strong ties” between members of internal groups, but also creates “weak ties” by attracting new members or facilitating cross-group interactions, thus expanding the network of social capital (17). Group physical activities in the community (e.g., square dancing, tai chi) create opportunities for face-to-face interaction among older adults, allowing participants to build empathy and trust through frequent interactions (44). This trust is at the core of social capital as defined by Robison et al. For older adults, good social relationships are an important source of happiness, and through the support and help of others, they are less likely to suffer from mental illness and more likely to feel a sense of well-being. Social capital indirectly increases participants’ subjective well-being by enhancing interpersonal trust, expanding interaction networks, and conveying emotional support (19). Older persons should therefore actively participate in community physical activity to gain more social capital, thereby strengthening social ties, improving social skills, promoting broad social participation and mutual trust, and thus enhancing their subjective well-being.

### 5.3 The moderating role of intergenerational support

In this study, the effects of both community physical activity and social capital on older adults’ subjective well-being were moderated by intergenerational support. The effects of community physical activity and social capital on older adults’ subjective well-being were enhanced under high levels of intergenerational support, which is similar to previous research that intergenerational support enhances the level of older adults’ subjective well-being (45).

This study demonstrates that intergenerational support moderates the effects of community physical activity on older adults’ subjective well-being. Specifically, the higher the level of intergenerational support received by older adults, the stronger the positive effect of community physical activity on older adults’ subjective well-being. Under the Chinese concept of filial piety, both emotional and financial support from children can contribute to the well-being of older adults (45). Good intergenerational relationships can also expand the social circle of the older adult and promote their participation in various community physical activities (46). From the perspective of financial support, children give more financial support to the older adult, which helps to improve their living conditions and provides resources for them to participate in community physical activities, such as purchasing clothing, equipment and other supplies, or paying for some sports club membership fees. This also conveys the love and filial piety of children for the older adult, in keeping with the traditional Chinese culture of “raising children to protect the older adult.” From the perspective of emotional support, children’s encouragement, affirmation and positive verbal support will make older people feel supported and recognized, and develop their sense of purpose and value, which can stimulate older people’s willingness to actively participate in physical activity, increase their self-confidence in exercise (47). In conclusion, children should take on the responsibility of caring for the older adult by giving them a certain amount of financial and emotional support and encouraging them to actively participate in community physical activity through various means, such as emotional response, accompanying participation and understanding support, in order to obtain common physical and mental health. Of course, the older adult themselves should also recognize the benefits of community physical activity to their physical and mental health and actively participate in community physical activities.

## 6 Limits

Although this study verified the multiple factors of the effect of community physical activity on the subjective well-being of older adults, there are still some limitations. First, this study is a cross-sectional study that does not provide a good explanation of the causal relationships among the variables. Second, the intergenerational support explored in this study only involves unidirectional children’s support for their parents; however, intergenerational support is bidirectional, and older adults can give intergenerational support to their children in addition to receiving intergenerational support from their children, which was not examined in this study. Third, the current study collectively referred to all sports programs in which older adults participate in the community as community physical activity without subdividing each program, and it is necessary for future research to conduct in-depth studies of a separate program. Finally, the current study only considered the mediating role of social capital and the moderating role of intergenerational support, and community physical activity affecting the subjective well-being of older adults may also be influenced by other factors, which should be continued to be explored in future studies.

## 7 Conclusion

This study examined the mediating role of social capital in the effect of community physical activity on older adults’ subjective well-being and the moderating role of intergenerational support. Findings suggest that community physical activity significantly and positively predicts older adults’ subjective well-being. Social capital partially mediated the relationship between community physical activity and older adults’ subjective well-being, and intergenerational support moderated the relationship between community physical activity and older adults’ subjective well-being. These findings emphasize that active participation in community-based physical activity can directly increase older people’s subjective well-being on the one hand, and indirectly increase well-being by increasing social capital on the other. In addition, good intergenerational relationships can lead to more active participation in community physical activity, which in turn affects the subjective well-being of older people.

## Data Availability

The raw data supporting the conclusions of this article will be made available by the authors, without undue reservation.
